# Description of the person-environment interaction: methodological issues and empirical results of an Italian large-scale disability assessment study using an ICF-based protocol

**DOI:** 10.1186/1471-2458-11-S4-S11

**Published:** 2011-05-31

**Authors:** Carlo Francescutti, Francesco Gongolo, Andrea Simoncello, Lucilla Frattura

**Affiliations:** 1 Italian WHO Collaborating Centre for the Family of International Classifications, Direzione Centrale Salute, Integrazione Socio Sanitaria e Politiche Sociali, Regione Autonoma Friuli Venezia Giulia, Udine, Italy

## Abstract

**Background:**

There is a connection between the definition of disability in a person-environment framework, the development of appropriate assessment strategies and instruments, and the logic underpinning the organization of benefits and services to confront disability.

**Methods:**

The Italian Ministry of Health and Ministry of Labor and Social Policies supported a three-year project for the definition of a common framework and a standardised protocol for disability evaluation based on ICF. The research agenda of the project identified 6 phases: 1) adoption of a definition of disability; 2) analytical breakdown of the contents of disability definition, so as to indicate as clearly as possible the core information essential to guide the evaluation process; 3) definition of a data collection protocol; 4) national implementation of the protocol and collection of approximately 1,000 profiles; 5) proposal of a profile analysis and definition of groups of cases with similar functioning profiles; 6) trial of the proposal with the collected data. The data was analyzed in different ways: descriptive analysis, application of the person-environment interactions classification tree, and cluster analysis.

**Results:**

A sample of 1,051 persons from 8 Italian regions was collected that represented different functioning conditions in all the phases of the life cycle. The aggregate result of the person-environment interactions was summarized. The majority of activities resulted with no problems in all of the A&P chapters. Nearly 50.000 facilitators codes were opened. The main frequent facilitators were family members, health and social professionals, assistive devices and both health and social systems, services and politics. The focus of the person-environment interaction evaluation was on the A&P domains, differentiating those in which performance presented limitations and restrictions from those in which performance had no or light limitations and restrictions. Communication(d3) and Learning and Applying Knowledge(d1) appeared as the more problematic A&P areas. Self Care(d5) was the domain in which facilitators were more effective in supporting functioning, suggesting that the Italian welfare system is mainly focused on providing care services for activities of daily living, jointly with the family. The cluster analysis was limited to those categories that were common to all age classes (38 categories out of 55). For a final representation, a solution with 6 clusters was chosen.

**Conclusions:**

An example is provided of how it is possible to plan empirical studies in which theoretical advances and operative goals on disability in a person-environment framework can support the definition of a research design, measurement strategies, and data analysis. The description of functioning and disability at population level is no more based on individual deficits or limitations. Personal profiles may be elaborated and groups created based on the characteristics of the person-environment interactions. Personal profiles may also be used as a “rationale” for defining personalized intervention programs.

## Background

The Italian Ministry of Health and Ministry of Labor and Social Policies supported a three-year project for the definition of a common framework and a standardised protocol for disability evaluation based on ICF. The project was carried out by the research team of the Italian WHO Collaborating Centre for the Family of International Classifications. Eight Italian regions and their officers were involved in the training of selected health professional teams, sample selection, and evaluation [1]. The assessment system, which was traditionally focused on impairments, was redesigned in order to preserve the continuity of a person-centred approach throughout the life span and the health and social service providers, keeping in mind individuals with multiple needs who have to negotiate with the health and social systems, both at national and regional level [2].

Basically, the idea was to define the technical premises necessary to:

• Recognize and evaluate the condition of disability, using common methodologies and instruments based on the person-environment interaction framework, across the network of health and social services and institutions;

• Allow interoperability and information sharing, on a common semantic base, between health and social information systems;

• Arrange the fragmented interventions and disability policies, at least from the point of view of the organization, management and information continuity, as components of a single personalized project of intervention embracing health and social systems as well as private resources (personal resources, family resources and local community resources).

In Italy, more than 3% of the total amount of resources allocated to the health and social sector are intentionally oriented to the support of functioning and personal empowerment [3]. Nevertheless, assessment criteria and eligibility criteria across the Regions are not easily comparable.

In the last 20 years, due to a process of devolution from the national Government of some health and social issues, the Italian Regions have developed a complex network of residential and home care facilities and health and social services for people with disability. Eligibility criteria, based on different and often non-comparable definitions of “not self-sufficient person” (dependent people), have been adopted at local level. This has led to the selection of various assessment tools and measurement instruments, ranging from the Katz Index [4] to more complex systems of evaluation, related in any case to the daily need for personal care. Globally, the Italian system is extended in coverage and in the range and types of benefits provided, but it appears fragmented and non-homogenous. Moreover, the risk of generating inequity is high and not controlled. The lack of a systemic view in the organization of the services implies that the person and his/her family have to became the real “system integrator” [5]. In many cases, this is extremely difficult and in some cases it results in an impossible task. The majority of benefits and services for persons with disability are not oriented by a logic of enhancing functioning and reducing disability but mainly to identify and compensate impairments.

### Disability as result of a person-environment relationship

The understanding and the establishment of a theory and praxis of a person-environment interaction in disability is just at its initial steps [6]. Defining disability as the result of an interaction between a person and the environment directly implies that disability is a systemic attribute. This connection is the foundation of the so called “social model” of disability insofar as the scenario that defines disability regards more actors than the individual, including the relationship with the social context [7-9]. The ICF interaction model [10], as noted by Fougeyrollas et al. [6], simply shows the links between the relevant “health components”, being not a representation of a person-environment interaction in causal terms. It allows to collect information related to health conditions, body functions and body structures, activity and participation, environment and personal factors, but it does not define the nature and characteristics of the relationships between the different components. At the same time, the UN Convention on Rights of Persons with Disability [11] defines the person with disability without describing what disability is. Although they provide an excellent conceptual framework, neither the ICF nor the UN Convention gives an operational definition of functioning and disability.

Although the general conceptual reference is common, there is not a unique language used to describe the person-environment interaction. Differences exist, between the approach of ecological developmental psychology [12] and that referred to as the “theory of systems” [13]. The “dynamic of the system” is also controversial. Sometimes it is ignored, sometimes the structure of a system and also its dynamic are described jointly in a single pattern. In this case, analytical schemas risk to be very misleading. In particular, the use of bidirectional arrows to define relationships that are different in nature and not temporally simultaneous, far from clarifying the characteristics of the person-environment interaction, simply underlines the “complexity” of the system and the trivial idea that “all influences all” [6]. As noted by Rogoff [14], the meaning of schemas and arrows is also not always explicit. Environment and persons are put separately in the same diagram, where they appear to be linked by arrows. But the environment and the person are not separate entities. Persons are literally immersed in their environment. Furthermore, oriented arrows should define a causal relationship and not simply an association between variables.

In this paper, an example is provided of how it is possible to plan empirical studies in which theoretical advances and operative goals can support, coherently, the definition of a research design, measurement strategies, and data analysis. The idea that these three issues have to progress together is strongly supported because of the interchange between fields of research.

## Methods

The research agenda of the Italian project identified 6 phases [1]:

1. adoption of a definition of disability;

2. analytical breakdown of the contents of disability definition, so as to indicate as clearly as possible the core information essential to guide the evaluation process;

3. definition of a data collection protocol;

4. national implementation of the protocol and collection of approximately 1,000 profiles;

5. proposal of a profile analysis and definition of groups of cases with similar functioning profiles;

6. trial of the proposal with the collected data.

### Definition of person with disability

#### a) The role of UN Convention

Article 1 of the UN Convention on the Rights of Persons with Disabilities was adopted as a reference definition for the Italian project. It stated that “ Persons with disabilities include those who have long-term physical, mental, intellectual or sensory impairments which in interaction with various barriers may hinder their full and effective participation in society on an equal basis with others”[11]. The use of the UN Convention as a guide in the process of disability assessment is well discussed in other articles within this issue of BMC Public Health, in particular in the work of Bickenbach [15]. Some peculiar aspects though have to be remarked: a) the interactive nature of disability is clear in the UN Convention and the choice to refer disability to permanent impairments is quite reasonable for its application in welfare policies; b) the UN Convention has been explicitly ratified by the Italian Parliament as a basis for the development and modification of the existing legislation;and c) a limit in the definition could be the emphasis on barriers, obscuring the role of facilitators and apparently making the definition quite similar to that of “handicap”.

#### b) The role of ICF

ICF describes disability as an umbrella term for impairments, activity limitations, and participation restrictions. It denotes the negative aspects of the interaction between an individual (with a health condition) and that individual’s contextual factors (environmental and personal factors) [10]. However, in ICF, barriers and facilitators have the same importance and therefore the umbrella term ‘functioning’ is used to describe the positive interaction between an individual and that individual’s contextual factors. In this study, the positive/negative interaction between a person and the environment was operationalized with the Activities and Participation (A&P) qualifiers.

### Contents of the evaluation and definition of a protocol for data collection

**Figure 1:**
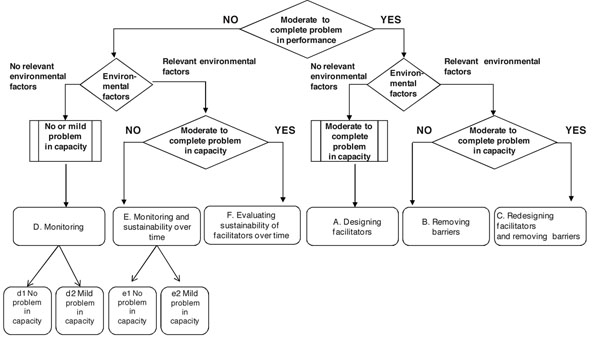
Person-environment interaction classification tree

As shown in Figure 1, the ICF model of disability suggested the definition of 8 macro groups of data to evaluate disability: 1) diagnosis (considered as a proxy for health conditions); 2) socio-demographic information; 3) person perception and evaluation of current needs (all considered useful to describe personal factors, without possibility to code them by ICF); 4) services and support provided by health and social systems; 5) drug therapy; 6) caregivers, informal support, caregivers’ burden (all considered useful to describe environmental factors as classified by ICF); 7) body functions and body structures (BFs and BSs); and 8) A&P in association with environmental factors (EFs), to describe functioning and disability according to the ICF definition.

The areas of life involved in functioning/disability evaluation were identified mapping the contents of the different UN Convention articles to ICF; they were referred, directly or indirectly, to a closed list of ICF A&P categories.

#### The focus of A&P evaluation

ICF A&P categories were coded using three qualifiers: the *performance* qualifier (P), which occupies the first digit position after the point, the *performance without assistance* qualifier (P1), which occupies the second digit position after the point, and the *capacity* qualifier (C ), which occupies the third digit position after the point.

According to ICF, the *performance* qualifier is the core of the evaluation and describes what an individual does in his or her current environment [10]. The *capacity* qualifier has been defined as the performance in a “modified environment” [3] and describes the “probable level” of functioning after removing the effect of environmental barriers or facilitators. The *performance without assistance* qualifier is an ICF optional qualifier that has been adopted to describe a special type of performance evaluating the “probable level” of functioning once the effect of the environmental factors related to support and relationship has been removed (chapter 3 of the EFs component in ICF).

Following the coding schema of ICF [10], proposed as a useful practice in the Australian ICF User’s guide [16], each ICF A&P item was coded using the three qualifiers, together with the EFs expressed with their own qualifiers. Thus, it was possible to map in detail all the significant elements of the person-environment interaction.

**Figure 2:**
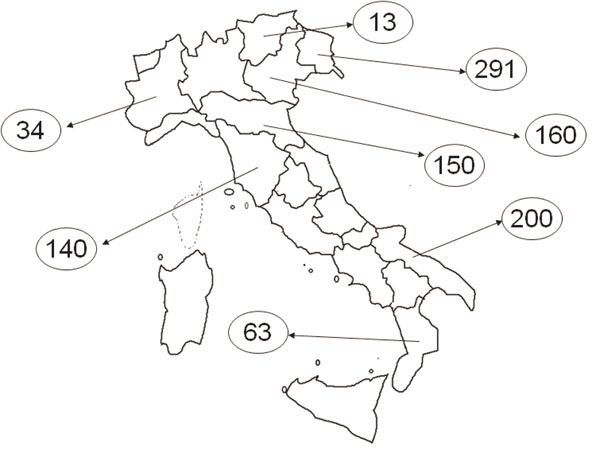
The research sample by region

To better describe the person-environment interaction, for each ICF A&P item, the information was stored in a matrix (Figure 2). While the A&P categories to be coded were those of a closed list, the categories of EFs were selected from the entire component. Furthermore, some environmental factors of the first domain were specified using a list of ISO9999 codes for assistive devices [17], also shown in the matrix .

The interaction was defined as negative every time that the assessed value of the first ICF A&P qualifier was the expression of a severe or complete difficulty; in contrast, the interaction was defined as positive every time that the assessed value of the first ICF A&P qualifier was the expression of none or mild difficulty.

#### The study population

A sample of 1,051 persons from 8 Italian Regions was evaluated using the ICF-based assessment protocol (see Figure 2). It was selected through a systematic random procedure, stratified by age class and gender using the list of persons applying for invalidity and handicap assessment at the local medical commissions in the year 2008.

### Data analysis

#### The person-environment interaction classification tree (PEIC tree)

The basic idea of the data analysis was to classify each ICF A&P item by evaluating the specific relationship between person and environment and using a “classification tree”, as described in a previous work [1] and updated in this one. The PEIC tree is depicted in Figure 1 and it shows six main classes of interactions (classes A to F).

The input to the PEIC tree was not the information on the person but the information on the person-environment relationship given by any A&P coded category together with the performance and capacity qualifiers. The process of classification distinguished A&P categories that described positive person-environment interactions from categories that described negative person-environment interactions. The distinction was made by considering the value of the performance qualifier. If it expressed a severe or complete difficulty, the item was processed on the right side of the “tree”, otherwise on the left.

#### The left side of the classification tree: positive person-environment interactions

In terms of person-environment relationship, the absence of limitations or restrictions in A&P, as shown by the P value = 0 or 1, may identify two main different conditions: one in which EPs do not exist (no limitation in capacity), and one in which EFs play a role in defining a positive interaction (capacity values describing moderate or severe limitation).

Using the PEIC tree, this general assumption may be more precisely described. The “D Class” interactions identify those A&P categories for which the assessed person does not have any significant difficulty in functioning (P value = C value, and they may be both = 0 or 1). These activities have to be monitored over time since the person and environmental factors may change, thus changing the effect of the balance between person and environment (Figure 1).

The “F class” and the “E class” identify different situations. The first one occurs when a person benefits, in specific A&P categories, of a system of facilitators allowing him/her to overcome limitations and restrictions (P value = 0 or 1 and different from C value). In some way, the person is in a position of compensation and has found a solution to his/her problems in specific A&P categories. The main question arising is related to the sustainability over time of the system of facilitators in terms of economic and human resources (F class). The “E class” can in turn identify two conditions: one in which the EFs act as facilitators (P value), and another one in which facilitators are present in absence of problems in capacity and in some way they seem to “over support” the person. In the first case, it is important to monitor and at least to evaluate sustainability over time of the system of facilitators (E2 class). In the second case, the opportunity to promote empowerment has to be investigated (E1 class).

#### The right side of the classification tree: negative person-environment interactions

If a moderate to complete limitation or restriction in A&P is ascertained, three possible situations may occur. In the first one, no facilitators or barriers (other than the lack of opportunities) are present to assure a good performance. The question arising is how to design facilitators providing an adequate support system for the person (A class). In the second situation, the person has to face barriers at any level of the environment and requires a project of “barriers removal” (B class)**.** In the third situation, barriers and facilitators do not effectively support a good performance. The question is how to redesign the system of facilitators and eventually how to remove barriers (C class).

#### Summarizing the person-environment interaction

To summarize the person-environment interaction, a matrix was realized in which the classes generated by classifying ICF A&P categories with the PEIC tree procedure were distributed so as to group A&P showing negative interactions and A&P showing positive interactions. The matrix was created both at individual level and at population level and was useful to provide detailed information on the concurrent presence of disability and functioning in the same individual or in the same population (describing disability as a continuum instead of as a social category) as well as to evidence how much disability is present in a population or in an individual (answering statistical questions as well as clinical or rehabilitative questions).

#### Grouping people with similar person-environment patterns

The data analysis was also oriented to identify groups of people that share common patterns of person-environment interaction. The output of the PEIC tree was used as input for a cluster analysis. A preliminary exploration of data was made using a standard clustering algorithm (K-Means). The input of the cluster analysis were, for each person, the counts of ICF A&P categories in each cell of the PEIC tree.

## Results

Aggregated data are presented focusing the attention on the use of ICF in describing A&P and EFs.

### Descriptive data

Table 1 shows the final sample. Table 2 shows the heterogeneity of diseases and health conditions in the study population. All the categories of diseases coded in ICD10 are present in the sample. Fifty-six point four percent of the cases had a psychiatric diagnosis, 50.4% a disease of the circulatory system, 36.8% a disease of the nervous system, and 20.5% a musculoskeletal health problem. On average, the selected cases had 3 coded diagnoses.

**Table 1:** The final sample by age class and gender

Age class	Male	Female
0-5	*125* (94)	*125* (65)

6-17	*125* (162)	*125* (110)

18-64	*125* (142)	*125* (162)

65-W	*125* (141)	*125* (175)

In italics the planned structure of the sample, in parenthesis the cases included in the data analysis

**Table 2:** Main diagnosis by main ICD10 chapters

ICD10 chapters	No. of ICD10 codes	% on total codes	% on total cases
Mental and behavioral disorders	583	19.0	56.4

Diseases of the circulatory system	521	17.0	50.4

Diseases of the nervous system	380	12.4	36.8

Diseases of the musculoskeletal system and connective tissue	305	9.9	29.5

Endocrine, nutritional and metabolic diseases	219	7.1	21.2

Neoplasms	187	6.1	18.1

Congenital malformations, deformations and chromosomal abnormalities	145	4.7	14.0

Symptoms, signs and abnormal clinical and laboratory findings, not elsewhere classified	124	4.0	12.0

Diseases of the ear and mastoid process	123	4.0	11.9

Diseases of the eye and adnexa	86	2.8	8.3

Diseases of the digestive system	79	2.6	7.6

Injury, poisoning and certain other consequences of external causes	75	2.4	7.3

Diseases of the genitourinary system	67	2.2	6.5

All remaining chapters	176	5.7	14.9

**Total**	**30.170**	**100.0**	

Table 3 shows the sample distribution in the ICF A&P domains by level of difficulty in performance and capacity. The best performance was in the Self-care domain, where effective support was provided, as expected. In contrast, the performance in the Mobility domain was critical.

**Table 3:** Sample distribution by level of difficulties in ICF A&P domains

ICF A&P domains	No moderate to complete difficulty in performance No. of cases (%)	At least one moderate to complete difficulty in performance (No. of cases)	No moderate to complete difficulty in capacity No. of cases (%)	At least one moderate to complete difficulty in capacity (No. of cases)	Total (No. of cases)
d1 Learning and applying knowledge	579 (55.1)	472	394 (37.5)	657	**1051**

d3 Communication	554 (52.7)	497	394 (37.5)	657	**1051**

d4 Mobility	354 (33.7)	697	276 (26.3)	775	**1051**

d5 Self care	969 (92.2)	82	314 (29.9)	737	**1051**

d6 Domestic life	437 (41.6)	614	228 (21.7)	823	**1051**

d7 Interpersonal interactions and relationships	637 (60.6)	414	523 (49.8)	528	**1051**

d8d9 Major life areas - Community, social and civic life	684 (65.1)	367	233 (22.2)	818	**1051**

In all A&P domains	139 (13.2)	912	50 (4.8)	1001	**1051**

In Tables 4a and 5, the distribution of environmental factors (facilitators and barriers) is given for each ICF A&P domain. An amount of 47.110 EFs were coded as facilitators in association with the list of A&P categories. The number of EFs coded as barriers was 2.753.

**Table 4:** Distribution of environmental factors across ICF A&P domains: facilitators

		ICF A&P domains						
	
ICF environmental factors domains		d1 Learning and applying knowledge	d3 Communication	d4 Mobility	d5 Self care	d6 Domestic life	d7 Interpersonal interaction and relationship	d8d9 Major life areas - Community, social and civil life
	
		No. of EF codes (%)	No. of EF codes (%)	No. of EF codes (%)	No. of EF codes (%)	No. of EF codes (%)	No. of EF codes (%)	No. of EF codes (%)
e1	Products and technology	1706 (18.5)	1164 (17.4)	2663 (24.7)	920 (11.7)	348 (9.6)	416 (9.7)	569 (12.3)

e2	Natural environment and human-made changes to environment	8 (0.1)	7 (0.1)	19 (0.2)	8 (0.1)	4 (0.1)	5 (0.1)	14 (0.3)

e3	Support and relationships	4730 (51.4)	3585 (53.5)	5232 (48.5)	5127 (64.9)	2673 (73.7)	2276 (53.3)	2724 (58.7)

e4	Attitudes	331 (3.6)	251 (3.7)	177 (1.6)	250 (3.2)	80 (2.2)	648 (15.2)	230 (5.0)

e5	Services, systems and policies	2423 (26.3)	1692 (25.3)	2689 (24.9)	1589 (20.1)	520 (14.3)	926 (21.7)	1106 (23.8)

**Total**		**9198** **(100)**	**6699** **(100)**	**10780** **(100)**	**7894** **(100)**	**3625** **(100)**	**4271** **(100)**	**4643** **(100)**

**Table 5:** Distribution of environmental factors across ICF A&P domains: barriers

		ICF A&P domains						
	
ICF environmental factors domains		d1 Learning and applying knowledge	d3 Communication	d4 Mobility	d5 Self care	d6 Domestic life	d7 Interpersonal interaction and relationship	d8d9 Major life areas - Community, social and civil life
	
		No. of EF codes (%)	No. of EF codes (%)	No. of EF codes (%)	No. of EF codes (%)	No. of EF codes (%)	No. of EF codes (%)	No. of EF codes (%)
e1	Products and technology	54 (13.6)	42 (14.0)	270 (38.8)	79 (24.8)	36 (17.1)	40 (9.3)	68 (16.9)

e2	Natural environment and human-made changes to environment	41 (10.4)	30 (10.0)	106 (15.2)	6 (1.9)	7 (3.3)	26 (6.1)	28 (7.0)

e3	Support and relationships	140 (35.4)	113 (37.5)	95 (13.6)	76 (23.8)	57 (27.0)	169 (39.5)	128 (31.8)

e4	Attitudes	62 (15.7)	59 (19.6)	57 (8.2)	56 (17.6)	46 (21.8)	155 (36.2)	84 (20.9)

e5	Services, systems and policies	99 (25.0)	57 (18.9)	168 (24.1)	102 (32.0)	65 (30.8)	38 (8.9)	94 (23.4)

**Total**		**396** **(100)**	**301** **(100)**	**696** **(100)**	**319** **(100)**	**211** **(100)**	**428** **(100)**	**402** **(100)**

Facilitators were concentrated in the first (Products and Technology: 7.786 codes), third (Support and Relationships: 26.347 codes) and fifth (Services, systems and policies: 10.945 codes) environmental factors domains (Table 4). The distribution of the facilitators had a peak in the ICF Mobility domain for Products and technology (24.7% of the facilitators) and a peak in the ICF Self-care (64.9%) and Domestic life (73.7%) domains for Support and relationships. Services, systems and policies were distributed more homogeneously across the ICF A&P domains, with a maximum in the ICF Learning and applying knowledge domain. The distribution of barriers was less polarized than that of facilitators. Relatively similar percentages of barriers were present in each A&P domain (Table 5).

### Application of the person-environment classification tree to the A&P categories

Table 6 shows the result of the application of the person-environment classification tree (Figure 1) to A&P categories based on the value of their qualifiers in association with EFs. Numerical values in the Table represent the percentage of categories of each A&P domain that was placed in a given classification tree class by the classificatory rules.

**Table 6:** Percentage of categories of ICF A&P domains by person-environment classification tree classes

ICF A&P domains								
**Classification tree classes**		**d1 Learning and applying knowledge**	**d3 Communication**	**d4 Mobility**	**d5 Self care**	**d6 Domestic life**	**d7 Interpersonal interaction and relationship**	**d8d9 Major life areas - Community, social and civil life**

**A**	**Designing facilitators**	14.0	16.0	11.2	0.8	0.1	0.2	0.2

**B**	**Removing barriers**	0.2	0.2	0.4	0.1	0.2	1.2	1.0

**C**	**Redesigning facilitators and removing barriers**	6.0	5.8	3.9	1.1	18.6	15.8	14.3

	**Total**	**20.2**	**22.0**	**15.5**	**2.0**	**19.0**	**17.2**	**15.5**

**D**	**Monitoring**	53.6	56.8	57.6	45.7	22.4	55.7	34.6

**D1**	*No difficulty in capacity*	47.0	49.4	49.3	44.6	19.9	50.7	32.3

**D2**	*Mild difficulty in capacity*	6.6	7.4	8.3	1.1	2.5	5.0	2.3

**E**	**Monitoring and evaluating sustainability over time**	13.0	9.4	8.2	19.1	12.8	13.4	16.1

**E1**	*No difficulty in capacity*	1.5	1.3	1.6	1.3	1.0	2.9	2.3

**E2**	*Mild difficulty in capacity*	11.5	8.1	6.6	17.8	11.8	10.5	13.8

**F**	**Evaluating sustainability of facilitators over time**	13.2	11.8	18.7	33.2	45.8	13.7	33.8

	**Total**	**79.8**	**78.0**	**84.5**	**98.0**	**81.0**	**82.8**	**84.5**

The first three rows of the Table (grey area) show the percentage of A&P categories characterized by a difficulty in performance. The highest percentage of categories with difficulty was in the Communication domain (22.0%),whereas the lowest in the Self Care domain (2.0%). The cases for which the problems in functioning were related to the presence of barriers only were relatively rare. In the ICF A&P domains Learning and applying knowledge, Communication, and Mobility, the difficulties in performance were mainly due to the lack of facilitators. In ICF domains Domestic life, Interpersonal interactions and relationship, and Major life areas - community, social and civil life, the difficulties were related to a mix of inefficacy of the existing facilitators and to the presence of barriers.

The second half of the Table shows the percentages of ICF A&P categories characterized by good performance.

The 45.8 % of categories in Domestic life domain, 33.8 % in Major life areas - community, social and civil life domain, and 3 3.2% in Self-care domain were classified in F class. A percentage ranging from 8.2% to 16.1% of categories regards situations in which a supporting environment allows an effective contrast to mild problems in capacity (E class in Figure 1). The percentage of A&P categories for which there is an association between no or mild problems in performance and capacity and no mention of specific barriers or facilitators ranges from 22.4% (Domestic life) to 57.6% (Mobility) (D class).

### From item classification to grouping of cases

The cluster analysis was limited to those categories that were common to all age classes (38 categories out of 55). For a final representation, a solution with 6 clusters was chosen. Table 7 presents an easily understandable pattern. Clusters 1 to 4 regard persons with very limited areas of difficulties in performance. From left to right, good performance was achieved through growing intensity and presence of facilitators. Cluster 4 had 18.4 categories on average (half of the total analyzed categories) for which the role of environmental factors in supporting performance was crucial. Clusters 5 and 6 represent persons with performance problems: in 30% and 54% of the A&P categories evaluated, respectively.

**Table 7:** Clusters of cases based on their similarities in person-environment interactions

Output of the person-environment interaction classification tree		Clusters					
	
		1	2	3	4	5	6
**A**	**Designing facilitators**	0.6	0.9	1.8	2.7	5.0	15.8

**B**	**Removing barriers**	0.1	0.1	0.2	0.1	0.2	0.0

**C**	**Redesigning facilitators and removing barriers**	0.4	0.3	1.3	1.6	5.9	4.6

	**Total A, B and C**	**1.1**	**1.3**	**3.3**	**4.4**	**11.1**	**20.5**

**D**	**Monitoring**	31.6	19.6	22.0	10.8	12.7	6.3

**E**	**Monitoring and evaluating sustainability over time**	3.2	10.5	4.3	5.2	2.8	1.1

**F**	**Evaluating sustainability of facilitators over time**	2.0	6.2	8.1	18.4	11.9	10.0

	**Total D, E and F**	**36.8**	**36.3**	**35.0**	**34.4**	**26.4**	**17.4**

## Discussion

The main objective of the Italian project was to propose a suitable solution for a common framework for disability as a systemic attribute, therefore incorporating an interactive definition of disability, an evaluation methodology, and proper instruments for the representation of this interaction. Considering the complexities of the tasks and the limits of the current research on the analyzed issues, the project tried to find a “practicable compromise” to be improved, integrated and refined over time. It aimed to propose a “prototype” incorporating a “new vision” that works even though it has to be improved .

Although the results are preliminary and the statistical procedure can be further refined, some considerations can be made:

a) it is possible to describe disability as a continuum according to a person-environment evaluation framework using ICF; and

b) it is possible to identify meaningful groups that could be used to define different profiles of intervention, or case-mix representation, no more based on personal characteristics but on the type of person-environment relationship.

Disability is multifactorial and complex [18]. If it is conceptualised using a complex model as ICF proposes and the UN Convention stresses, it is not possible to describe it in a single measure [19]. Mainly due to this initial state of the theorization, the assessment of disability in a systemic framework is also quite a difficult task [9]. The traditional approach to disability assessment is distant from that of an interactive model, as well documented by Rust & Smith [20]; in order to guarantee the comparability of results, common disability assessment instruments are poor in defining the conditions and characteristics of the environment in which the evaluation has to be realized. In contrast, in an interactive model, there is no more room for the idea of measuring disability as “an intrinsic characteristic” of the person and the environment plays an important role. This study shows that a conceptual refinement of the constructs and concepts guiding the definition of the disability assessment process, particularly the way in which the environment is taken into consideration, is possible and allows to read the role of environment in the functioning of persons with bad health conditions. As Salvador proposed [21], we could adopt the concept of environmental dependence to describe the situation of people whose functioning is classified by the person-environment interactions classification tree.

The possibility of summarizing the person-environment interaction allows us to consider the individual scenario as a base for intervention. It is not enough, of course, to make a decision but it could be used as a common and shareable reference for defining potential areas of intervention within a systemic framework and with a specific orientation to the dynamic of the system. Furthermore, the individual scenario represents a reference for monitoring the situation over time and evaluating the impact of interventions at personal and contextual level. The aggregated scenario instead is an interesting perspective to monitor the health status of the population and, at the same time, the efficacy of disability policies and their heterogeneous impact on different areas of human life. As underlined earlier, the sample cannot be considered representative of a specific population of persons with disability. Having this in mind, it is interesting to note that the representation of A&P data associated with EFs gives an immediate view of the disability profiles at aggregate level, distinguishing between areas of relatively good performance and good capacity, areas of good performance in the presence of effective and reactive environments, and areas with “open problems” and “persisting difficulties”.

The descriptive data show the heterogeneous presence of the environment in the different A&P domains. The fact that Self Care is better supported than Mobility can be explained by a traditionally consolidated attention to an area that refers more directly to health and social care.

The great difference between facilitators and barriers, taking into account the overall amount of these coded factors, may be due to the nature of the studied population, which experiences severe functioning problems within a complex system of supporting interventions.

As seen in the premises, the UN Convention on rights of people with disability itself takes into consideration only barriers. In addition to that, the way in which the Italian system is organized, could be also considered. In Italy the services are traditionally provided by the National and Regional Health systems and therefore considered as granted by the citizens that are not used to an “insurance” logic. In this sense, the health services provided by the Public health systems are considered as barriers only when they are not efficiently working. Furthermore, the evaluators that have made the assessments and compiled the protocols in this national project belong to the same health system that they were evaluating and are therefore not used to consider themselves as part of the environment surrounding the evaluated person.

These factors have all to be considered in order to understand the possible difficulties in detecting and evaluating the role and importance of barriers in everyday life of persons with disability.

With regard to the distribution of the environmental factors, the peak of Products and technology registered in the Mobility area is probably due to the simplicity of detecting assistive devices as a facilitator. Moreover, in the Italian system, Support and relationships are a strategic resource in any A&P domain and cover, in each domain, the majority of the specific A&P evaluated areas.

## Conclusions

An example is provided of how it is possible to plan empirical studies in which theoretical advances and operative goals on disability in a person-environment framework can support the definition of a research design, measurement strategies, and data analysis. The description of functioning and disability at population level was no more based on individual deficits or limitations. From a methodological point of view, the key problem to maintain the systemic thinking in the assessment procedure and capture the result of a person-environment interaction in data, figures and numbers was faced with. The idea of establishing a new “generation” of measures starting from ICF is extremely interesting. Nevertheless, thinking of functioning and disability as a continuum in ICF is misleading, suggesting a latent unidimensional construct that can be measured using classical psychometric procedures, ideally a sort of number that magically can express all the information related to functioning and disability.

The role of a person-environment perspective in reforming disability policies has been well highlighted in scientific works and in planning documents [14]. This implies that disability should be always considered as a dynamic process in which it is possible to organize a personalized program of interventions. Further studies are needed to describe personal profiles and groups based on the characteristics of the person-environment interactions to be used as a “rationale” for redefining welfare eligibility criteria and planning personalized intervention programs.

## Competing interests

The authors declare that they have no competing interests.
